# miR-30d-5p: A Non-Coding RNA With Potential Diagnostic, Prognostic and Therapeutic Applications

**DOI:** 10.3389/fcell.2022.829435

**Published:** 2022-01-27

**Authors:** Qinlu Zhao, Xin Yuan, Lian Zheng, Miaomiao Xue

**Affiliations:** ^1^ Department of Infectious Diseases, The First Affiliated Hospital of Zhengzhou University, Zhengzhou, China; ^2^ Department of Oral and Maxillofacial Surgery, The First Affiliated Hospital of Zhengzhou University, Zhengzhou, China; ^3^ Department of General Dentistry, The First Affiliated Hospital of Zhengzhou University, Zhengzhou, China

**Keywords:** MiR-30d-5p, human cancer, cancer therapy, tumor progression, prognosis

## Abstract

Cancer is a great challenge facing global public health. Scholars have made plentiful efforts in the research of cancer therapy, but the results are still not satisfactory. In relevant literature, the role of miRNA in cancer has been widely concerned. MicroRNAs (miRNAs) are a non-coding, endogenous, single-stranded RNAs that regulate a variety of biological functions. The abnormal level of miR-30d-5p, a type of miRNAs, has been associated with various human tumor types, including lung cancer, colorectal cancer, esophageal cancer, prostate cancer, liver cancer, cervical cancer, breast cancer and other types of human tumors. This reflects the vital function of miR-30d-5p in tumor prognosis. miR-30d-5p can be identified either as an inhibitor hindering the development of, or a promoter accelerating the occurrence of tumors. In addition, the role of miR-30d-5p in cell proliferation, motility, apoptosis, autophagy, tumorigenesis, and chemoresistance are also noteworthy. The multiple roles of miR-30d-5p in human cancer suggest that it has broad feasibility as a biomarker and therapeutic target. This review describes the connection between miR-30d-5p and the clinical indications of tumors, and summarizes the mechanisms by which miR-30d-5p mediates cancer progression.

## Introduction

Cancer remains an intractable disease worldwide, owing to the fact that its causes are not fully understood, and tumors are often found at an advanced stage, which makes treatment arduous (Siegel et al., 2019; Wu et al., 2019). Several methods have been conceived to treat malignant tumors, such as surgery, radiation, chemotherapy, immunotherapy, or photodynamic therapy (Mun et al., 2018; Zehnder et al., 2018). In particular, cancer immunotherapy has expanded the scope of tumor-targeted therapies (Van Den Bulk et al., 2018). In recent years, cancer therapy has evolved from relatively nonspecific cytotoxic drugs to selective, mechanism-based approaches (Vanneman and Dranoff, 2012). However, the drug resistance that cancer cells acquire during treatment makes the treatment less effective (Cui et al., 2018). Epigenetic abnormalities have been shown to be associated with cancer progression and have been the focus of some researches (Qu et al., 2013; Kanwal et al., 2015). In addition, some of the above treatments are only suitable for patients diagnosed at an early stage (Anwanwan et al., 2020). Timely diagnosis is therefore crucial, nevertheless, it is not frequently achieved in many cancers, such as liver and gastric cancer (Song et al., 2017). There is growing evidence that cancer screening assists with the reduction of cancer-related morbidity and mortality (Loud and Murphy, 2017). Thus, finding biomarkers of malignancy is pivotal for prompt diagnosis and subsequent treatment.

Non-coding RNAs are a class of RNAs that do not participate editing proteins and can be divided into three categories according to length. One is less than 50 nt, including microRNA, siRNA, piRNA, etc. The second is 50 nt to 500 NT, including rRNA, tRNA, snRNA, snoRNA, SLRNA, SRPRNA, and so on. The third is greater than 500 nt (Ma et al., 2013; Cech and Steitz, 2014; Hombach and Kretz, 2016; Paskeh et al., 2021). As miRNA sponges, circRNAs inhibit the activity of miRNAs and regulate gene expression (Panda, 2018; Yu and Kuo, 2019). And the regulation of lncRNAs and miRNAs have also been reported in a variety of cancers (Dong et al., 2019; Xu et al., 2019; Ghafouri-Fard et al., 2021). MiRNAs, a kind of endogenous non-coding RNAs consisting of 21-23 nucleotide sequences, participate in the regulation of gene expression (Zamani et al., 2020; Mirzaei et al., 2021a). They control post-transcriptional regulatory factors of gene expression and the translation process of target mRNAs via attachment of mRNA molecules at the 3′-UTR bases, thus reducing transcription of proteins (Lagos-Quintana et al., 2001; Bartel, 2004; Pu et al., 2019; Alizadeh-Fanalou et al., 2020). In addition, miRNAs bind to the 5′-UTR of mRNAs, which is a path to reduce protein transcription (O'Brien et al., 2018; Ashrafizadeh et al., 2021). MiRNAs were shown to be involved in cell proliferation (Cai et al., 2009; Wang et al., 2019b), differentiation (Shi et al., 2016; Salunkhe and Vaidya, 2020), apoptosis (Zhang et al., 2019) and other cellular activities in tumors (Mishra et al., 2016; Mirzaei et al., 2021b). In addition, There are mRNAs, miRNAs and ncRNAs in the lumen of exosomes, and exosomes fuse with cells to regulate recipient cells (Momen-Heravi et al., 2014; Zhang et al., 2015). The role of exosome miRNAs in tumors have been gradually revealed (Ashrafizaveh et al., 2021). For example, Li et al. confirmed the influence of dysregulation of exosome miRNAs on hepatocellular carcinoma and discovered the clinical significance of exosome miRNAs in the diagnosis of hepatocellular carcinoma (Li et al., 2018). Overall, the abnormalities of miRNAs have been associated with the occurrence and development of numerous diseases, especially human cancers (McGuire et al., 2015; Annamareddy and Eapen, 2017).

MiR-30d-5p is located on chromosome 8q24.22, and the genetic symbol is SLC7A5 (Li et al., 2017; Zhang et al., 2017), and is regulated by LINCRNA in a variety of cancers. The microRNA Cluster was miR-30 b/d, and the seed family of miR-30d-5p included miR-30abcdef/384–5p (Li et al., 2017). MiR-30d-5p is regulated by LncRNA PVT1 in gallbladder cancer (Yu et al., 2018), LncRNA SOX2-OT/miR-30d-5p is associated with the progression of non-small cell lung cancer (Chen et al., 2021), and LINC00284 to miR-30d-5p regulation is associated with the development of thyroid cancer (Hu C. et al., 2021). Growing evidences have highlighted the influence of miR-30d-5p on cell activities and its role in the occurrence and development of different cancer types, therefore, it could serve as a novel biomarker of diagnosis. For example, miR-30d-5p was targeted to inhibit cell activity in non-small cell lung cancer (Kranjc et al., 2020), and was also involved in the construction of prostate cancer diagnosis and a disease stratification model (Song et al., 2018). MiR-30d-5p was also reported to participate in the progression in esophageal squamous cell carcinoma (Zhu et al., 2017). Several studies demonstrated the connection between the dysregulation of tumor progression and miR-30d-5p, which was therefore considered as a potentially pivotal tumor biomarker.

This review aims to provide additional clues for future research on miR-30d-5p, and consists of two parts: the first one describes the connection between miR-30d-5p and clinical processes in different types of cancer, and the other summarizes the regulatory mechanisms involving miR-30d-5p.

## Expression of miR-30d-5p and Clinical Characteristics of Multiple Human Cancers

Growing evidence have revealed miR-30d-5p is likely to be related to clinical features. The following is an overview of the association between different types of cancers and miR-30d-5p. A summary of miR-30d-5p expression and cancer-related clinical indicators is provided in Table 1.

**TABLE 1:** The relationship between miR-30d-5p expression and clinical characteristics in multiple cancers.

Cancer type	Property	Studied species and biomaterial	Expression	Prognosis	Number of cases	Refs
Lung cancer	Suppressor	Human tumorous tissue	Down	Favorable	24	Hosseini et al. (2018)
	Oncogene	NSCLC cell lines and body fluid	Down	Poor	—	Li et al. (2017)
	Suppressor	Human NSCLC tissue and cell lines	Down	Favorable	80	Zeng et al. (2020)
	—	HEK 293T cells	Down	—	—	Chen et al. (2015)
	—	Human NSCLC tissue and cell lines	Down	—	—	Gao et al. (2018)
	Suppressor	Human NSCLC tissue	Down	Favorable	81	Zhang et al. (2017)
	Suppressor	Human LUSC cell lines	Down	Favorable	—	Qi et al. (2021)
	Suppressor	Human LUAD tissue and cell lines	Down	Favorable	20	Zhao et al. (2021)
	Suppressor	Human NSCLC cell lines	Down	—	—	Chen et al. (2021)
Gallbladder carcinoma	Suppressor	Human GBC tissue and cell lines	Down	Favorable	53	He et al. (2018)
Cholangiocarcinoma	Oncogene	Human bile and serum	Up	Poor	106	Han et al. (2020a)
Rectal cancer	—	Human CRC tissue and cell lines	Down	—	—	Bjørnetrø et al. (2019)
Colon cancer	—	Human tumorous tissue	—	—	396	Jacob et al. (2017)
	Suppressor	Human tumorous tissue and cell lines	Down	Favorable	60	Yu et al. (2018)
Prostate cancer	Suppressor	Human PCa tissue and PC cell lines	Down	Favorable	40	Song et al. (2018)
	—	Human PCa cell lines	Down	—	—	Kumar et al. (2016)
	—	Human seminal fluid and PCa cell lines	Down	Favorable	24	Barceló et al. (2020)
Esophageal squamous cell carcinoma	—	Human serum	Up	Poor	30	Zhu et al. (2017)
	Suppressor	Human ESCC tissue and cell lines	Down	Favorable	144	Guo et al. (2021)
	Suppressor	Human ESCC tissue and cell lines	Down	Favorable	64	Wang et al. (2020)
Ovarian cancer	Suppressor	The meta-analysis	Down	Favorable	—	Shi et al. (2018)
Cervical cancer	—	Human tumorous tissue	Down	Favorable	121	Zheng et al. (2019)
Breast cancer	—	Human tumorous tissue	Down	Favorable	96	Kunc et al. (2020)
Renal cell carcinoma	Suppressor	Human RCC tissue and cell lines	Down	Favorable	25	Liang et al. (2021)
Pancreatic cancer	Suppressor	Pancreatic cancer cell	Down	Favorable	—	Zheng et al. (2021)
Osteosarcoma	Oncogene	Human cell line HFOB1.19	Up	Poor	—	Hu Y. et al. (2021)
Thyroid cancer	Suppressor	Cell lines	Down	—	—	Hu C. et al. (2021)
Hepatocellular carcinoma	Suppressor	Human HCC tissue	Down	Favorable	36	Yu et al. (2019)
	Suppressor	Human HCC tissue and cell lines	Down	Favorable	25	Zhuang et al. (2019)

### Lung Cancer

The global incidence of lung cancer is extremely high. Pathologically, lung cancer can be divided into small cell lung cancer (SCLC) and non-small cell lung cancer (NSCLC), and the vast majority of lung cancer patients are diagnosed with the latter (Chen et al., 2014). Lung squamous cell carcinoma (LUSC) and lung adenocarcinoma (LUAD) are the common types of NSCLC, which is considered as clinically the most commonly encountered type of lung tumor (Kinoshita et al., 2015; Goodwin et al., 2017; Gelatti et al., 2019). Despite the premise of continuous exploration, the prognosis of this disease is poor, and its 5-year survival rate is unsatisfactory (Garon et al., 2019). According to relevant reports, the ectopic level of miR-30d-5p impeded tumor progression via inhibiting the invasion and motility of tumor (Chen et al., 2015). By targeting CCNE2, miR-30d-5p was shown to have an anticancer role (Li et al., 2017; Hosseini et al., 2018). More importantly, since the expression level of miR-30d-5p in tumors clearly differed from that in surrounding tissues, the expression of miR-30d-5p in tumor tissue decreased significantly (Zhao et al., 2021). Therefore, it was considered as a possibly important factor connected with the clinical diagnosis and treatment of NSCLC (Zhang et al., 2017; Gao et al., 2018). Interestingly, the content of miR-30d-5p varied between different grades of NSCLC, indicating that miR-30d-5p was associated with the occurrence and progression of NSCLC (Xue et al., 2021; Zhao et al., 2021). In addition, Qi et al. indicated that the expression of miR-30d-5p in LUSC cells was significantly downregulated, and the proliferation, migration and invasion of tumor cells were subsequently reduced, indicating that miR-30d-5p had a clear antitumor effect (Qi et al., 2021). Luciferase experiments also verified the targeted binding of miR-30d-5p with DBF4’s 3′-UTR region, which inhibited various physiological functions of cancer cells (Qi et al., 2021).

### Cholangiocarcinoma (CCA) and Gallbladder Carcinoma (GBC)

Cholangiocarcinoma (CCA) is a remarkable type of hepatic malignancy, whose incidence has been on the rise year by year in recent decades (Rizvi and Gores, 2013; Labib et al., 2019). Many patients still face a poor prognosis even after effective surgery (Esnaola et al., 2016; Oliverius et al., 2019). Studies have validated that miR-30d-5p shows an increasing trend in CCA; its expression level was markedly higher than in other benign diseases (Han et al., 2020a). Compared with other miRNAs, miR-30d-5p had higher specificity and sensitivity to distinguish benign and malignant diseases of the biliary tract. Therefore, miR-30d-5p is likely to be regarded as a potential biomarker for CCA (Han et al., 2020a). Gallbladder carcinoma is a common biliary tract cancer in China (Gomes et al., 2020; Murimwa et al., 2021). Lactate dehydrogenase A (LDHA) is found in many tissues and cells of the human body, and was found capable of facilitating the Warburg effect (Pathria et al., 2018) to produce lactic acid and ATP under aerobic conditions. He et al. stated that miR-30d-5p was often lowly expressed in GBC as opposed to the expression of LDHA. When miR-30d-5p had decreased expression, the survival rate of patients was also reduced. In addition, miR-30d-5p was overexpressed by targeting LDHA to reduce glycolysis in malignant tumors, and thus inhibited cancer development (He et al., 2018). In both gallbladder cancer and bile duct cancer, expression levels of miR-30d-5p were different from those in healthy tissues, which was worthy of further study in subsequent experiments.

### Colorectal Cancer

While the overall incidence of colorectal cancer has declined in recent years (Akgül et al., 2014), the number of patients below 50 years of age has rather been on the rise, which should be highly concerning (Haraldsdottir et al., 2014). One study linked miR-30d-5p with carcinogenesis in colon cancer through the long non-coding RNA, PVT1 (Patel and Ahnen, 2018). Specifically, PTV1 prevented miR-30d-5p from exhibiting its own function, resulting in the increased expression of the downstream factor RUNX2, a novel oncogene (Patel and Ahnen, 2018). The LASSO regression analysis revealed a 16-miRNA signature, including miR-30d-5p, which was then considered as an effective prognostic biomarker for stage 2 and 3 colorectal cancer, indicating the future direction of research on therapeutic targets for colorectal cancer (Jacob et al., 2017). Furthermore, miR-30d-5p was regarded as an indicator of cellular hypoxia in rectal cancer, which might promote cancer metastasis to specific organs (Bjørnetrø et al., 2019). Overall, miR-30d-5p is important for the diagnosis and treatment of colorectal cancer, but more clinical data are needed to support it.

### Esophageal Cancer

Esophageal cancer is a common malignancy with high morbidity and mortality due to its rapid development and invasiveness (Huang and Yu, 2018), while its early diagnosis is challenging and has poor prognosis (Bollschweiler et al., 2017; Short et al., 2017). In one study, the serum miR-30d-5p levels were significantly increased in esophageal squamous cell carcinoma patients, but they evidently declined after surgery. Additionally, miR-30d-5p could be associated with the TNM staging of esophageal cancer, suggesting its capability to become a potential biomarker in scientific research and subsequent clinical work (Zhu et al., 2017; Wang et al., 2020). Wang et al. indicated that LOC440173 could weaken the proliferation and invasion of malignant tumors though sponging miR-30d-5p (Wang et al., 2020). In other words, miR-30d-5p interacted with other molecules to influence the clinical indications of esophageal cancer. Additionally, Guo et al. showed that miR-30d-5p was decreased in esophageal cancer tissues, while its upregulation inhibited cancer progression (Guo et al., 2021). Although the mechanism of miR-30d-5p in esophageal cancer had been proven, more data are needed to support whether miR-30d-5p can be applied to clinical work.

### Prostate Cancer (PCa)

Prostate cancer is a common malignancy in males, with its incidence increasing with age (Grozescu and Popa, 2017). The course of the disease is slow, however, the mortality risk is considerable (Chang et al., 2014; Srougi et al., 2017; Teo et al., 2019). One report indicated that the negative regulation of NT5E in the tumor via miR-30d-5p tended to inhibit prostate cancer progression (Song et al., 2018). Specifically, the reduction of miR-30d-5p had a fundamental impact on the proliferation and migration of PCa cells. In addition, NT5E gene methylation has been shown to be associated with cancer (Lo Nigro et al., 2012; Jeong et al., 2020). A recent study reported that, during the development of the abnormal expression of PCa, related miRNA was applied to the seminal plasma, and the level of miR-30d-5p indicated the prognosis of prostate cancer, that is, its downregulation predicted poor prognosis (Barceló et al., 2020). The [PSA + miR-30d-5p + miR-93–5p] and [PSA + miR-30d-5p] models were also used for the prediction and diagnosis of prostate cancer (Barceló et al., 2020). Notably, when miR-30d-5p was upregulated, the androgen receptor activity was lower, and these two factors were often reversed (Kumar et al., 2016). Moreover, the level of miR-30d-5p expression was abnormal in chronic prostatitis (Chen et al., 2018), suggesting that miR-30d-5p could be considered a biomarker in benign and malignant prostate diseases.

### Other Cancers

In liver cancer, miR-30d-5p expression was associated with the cancer cell migration rate and patient survival rate, and thus considered useful for guiding the treatment of advanced liver cancer (Yu et al., 2019). In hepatocellular carcinoma (HCC), the target of miR-30d-5p, glycine decarboxylase (GLDC) (Zhuang et al., 2019), was closely related to the prognosis of HCC patients, and was considered as a separate factor for analysis in all probability. In female reproductive system tumors, namely ovarian cancer, a high expression of miR-30d-5p was able to attain better prognosis (Shi et al., 2018). In cervical cancer (Zheng et al., 2019), miR-30d-5p had the potential as an extraordinary diagnostic biomarker not only for invasive screening of tumors, but also their precursors. What is more, miR-30d-5p showed a diminished level in breast cancer (Kunc et al., 2020). miR-30d-5p also functioned to impede cell activity through the downstream factor ATG5 in renal cell carcinoma (Liang et al., 2021). One study suggested that miR-30d-5p contributed to the construction of pancreatic cancer regulatory network, thus providing a solution for the treatment of pancreatic cancer (Zheng et al., 2021). In addition, miR-30d-5p was validated to be associated with the occurrence and development of osteosarcoma (Hu Y. et al., 2021). miR-30d-5p was down-regulated in thyroid cancer and impeded tumor development through competitive binding with LINC00284 (Hu C. et al., 2021). Overall, in the cancers described above, miR-30d-5p had clinical significance and was nominated as a potential biomarker for research.

## The Functional Roles of miR-30d-5p in Cancers

miR-30d-5p generally exerts its effects through molecular mechanisms, such as those related to cancer cell proliferation, and some upstream and downstream targets. In addition to the above discussion on the connection between miR-30d-5p expression and clinical pathological features in various tumors, this paper reviews the molecular mechanisms that have been associated with miR-30d-5p below (Table 2).

**TABLE 2:** The biological functions and molecular mechanisms of miR-30d-5p.

Cancer type	Property	Functions	Genes/proteins/pathways	Refs
Lung cancer	Suppressor	Cell proliferation and motility inhibitor	Targeting CCNE2	Chen et al. (2015)
	Suppressor	Cell proliferation, migration, and invasion inhibitor	Targeted by POU3F3	Zeng et al. (2020)
	Suppressor	Cell proliferation, migration, and invasion inhibitor	Targeting DBF4	Qi et al. (2021)
	Suppressor	Cell proliferation, invasion and stemness inhibitor	circCD151/miR-30d-5p/GLI2 axis	Zhao et al. (2021)
	Suppressor	Malignant progression inhibitor and immune escape	LncRNA SOX2OT/miR-30d-5p/PDK1 driving PD-L1 through the mTOR signaling pathway	Chen et al. (2021)
Gallbladder carcinoma	Suppressor	Cell apoptosis promoter, and migration inhibitor	Targeting LDHA	He et al. (2018)
Rectal cancer	Suppressor	Organ-specificity of metastasis inhibitor	—	Bjørnetrø et al. (2019)
Colon cancer	Suppressor	TNM	—	Jacob et al. (2017)
	Suppressor	Cell proliferation, metastasis and lymph node inhibitor, tumor stage	LncRNA PVT1/miR-30d-5p/RUNX2 axis	Yu et al. (2018)
Prostate cancer	Suppressor	Cell proliferation, and migration inhibitor	Targeting NT5E	Song et al. (2018)
	Suppressor	Cell proliferation inhibitor	AR (Androgen receptor) regulating	Kumar et al. (2016)
Esophageal squamous cell carcinoma	Suppressor	Cell proliferation, migration, invasion inhibitor, and EMT inhibitor	LOC440173/miR-30d-5p/HDAC9 axis	Wang et al. (2020)
	Suppressor	Cell proliferation and invasion inhibitor, EMT inhibitor	Sponged by DDX11-AS1, regulating SNAI1/ZEB2 and Wnt/β-catenin pathway	Guo et al. (2021)
Renal cell carcinoma	Suppressor	Cell proliferation inhibitor, and autophagy promotor	Targeting ATG5	Liang et al. (2021)
Pancreatic cancer	Suppressor	Metastasis	miR-30d-5p/GJA1 CTNNA1, CTNNB1, CTNND1	Zheng et al. (2021)
Osteosarcoma	Oncogene	Cell migration, invasion, proliferation inhibitor, apoptosis promotor, and EMT	Regulating SOCS3/JAK2/STAT3 pathway	Hu Y. et al. (2021)
Thyroid cancer	Suppressor	Tumorigenesis	LINC00284/miR-30d-5p/ADAM12 regulating Notch signaling pathway	Hu C. et al. (2021)
Hepatocellular carcinoma	Suppressor	Cell migration inhibitor	—	Yu et al. (2019)
	Suppressor	Autophagy	Targeting GLDC	Zhuang et al. (2019)

### Cell Proliferation

**FIGURE 1:**
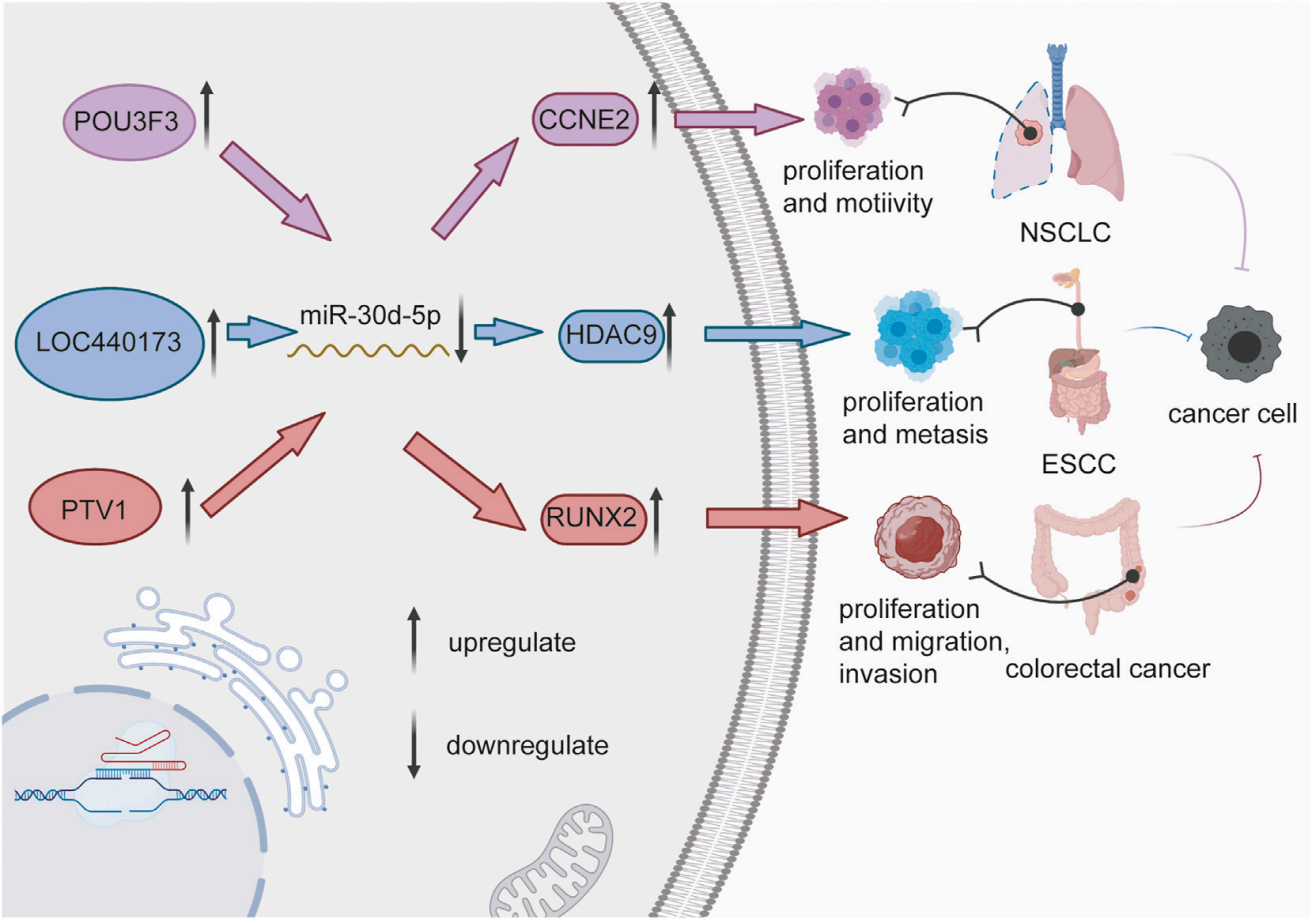
LncRNAs (POU3F3, LOC440173, and PTV1) sponge miR-30d-5p, thereby increasing the expression of downstream factors (CCNE2, HDAC9, and RUNX2), which in turn facilitate the activity of cancer cells, and promote the development of non-small cell lung cancer, esophageal squamous cell carcinoma and colorectal cancer.

In tumors, cell proliferation is obviously increased and is in close connection with cancer progression. The flow cytometry analysis revealed that miR-30d-5p overexpression gave rise to cell cycle arrest at the G0/G1 phase in prostate cancer (Song et al., 2018). miR-30d-5p directly targeted CCNE2 to impede tumor cell activity in NSCLC (Chen et al., 2015). Furthermore, in NSCLC cell lines, the overexpression of POU3F3 led to the negative regulation of miR-30d-5p to facilitate the proliferation of cancer cells. However, for cells with only POU3F3 overexpressed, the cell proliferation activity was much lower than that of cells with the deregulation of both. POU3F3 (Zeng et al., 2020) acted as an upstream regulator of miR-30d-5p to control CCNE2 downstream in NSCLC (Chen et al., 2015). Thus, the POU3F3/miR-30d-5p-CCNE2 signal was likely to be a new signaling pathway in NSCLC (Figure 1). In addition, one study reported that DBF4, a downstream gene of miR-30d-5p (Lankhaar et al., 2021), was overexpressed to activate the proliferation of cell lines in lung squamous cell carcinoma.

**FIGURE 2:**
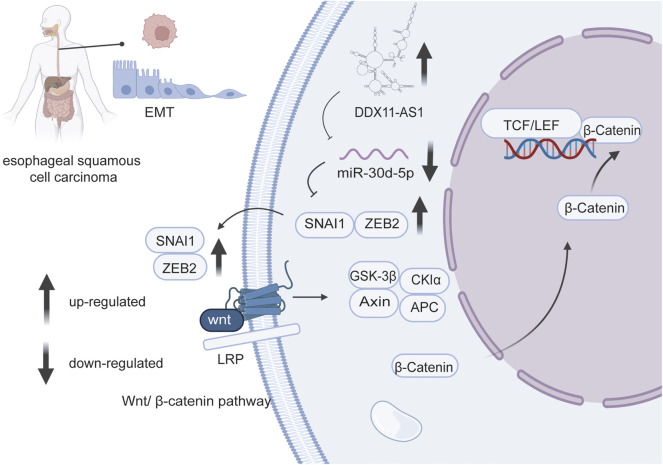
LncRNA DDX11 antisense RNA one promotes EMT process of esophageal squamous cell carcinoma by sponging miR-30d-5p to regulate SNAI1/ZEB2expression and Wnt/β-catenin pathway.

miR-30d-5p was also mentioned in connection with cell proliferation in other human cancers. In vitro studies, LOC440173 played a positive regulatory role in the development of esophageal cancer (Wang et al., 2020). The level of HDAC9, the target gene of miR-30d-5p, had the same trend as LOC440173 (Wang et al., 2020). The up-regulation of miR-30d-5p had a blocking effect on the proliferation of esophageal cancer cells, and this effect had also been verified in the invasion of cancer cells (Guo et al., 2021) (Figure 2). Compared with thymidine kinase 1 (TK1), a key enzyme in cell proliferation, miR-30d-5p had a higher sensitivity and specificity to the abnormal proliferation of esophageal squamous cell carcinoma cells (Zhu et al., 2017). Moreover, miR-30d-5p overexpression reduced the level of ATG5 (Liang et al., 2021), thus obviously inhibited the proliferation of tumor cells in renal cell carcinoma, and slowed the transition from the G1 to the S phase of the cell cycle. Furthermore, the high expression of miR-30d-5p weakened granuloma cell proliferation via targeting Smad2 (Yu and Liu, 2020). Wu et al., 2018 established that DGCR5 upregulated the level of Runx2 via miR-30d-5p and induced the osteoblast differentiation in human mesenchymal stem cells. In general, miR-30d-5p has been strongly connected with cell proliferation in various cancers.

### Cell Motility

In tumors, cellular activity signifies the invasion and metastasis of cancer cells. PVT1 directly regulates the progression of colorectal cancer via the miR-30d-5p/RUNX2 axis (Figure 1) (Yu et al., 2018). Experiments have shown that the silencing of PTV1 induced miR-30d-5p regulation, which remained a significant suppression impact on the invasion of colorectal cancer. The high expression of miR-30d-5p significantly inhibited the migration of PCa cells, while the overexpression of NT5E (Rodemer-Lenz, 1989) reduced the impact of miR-30d-5p on the invasiveness of PCa cells. Similarly, the elevated expression of CCNE2 boosted the invasion, and migration of NSCLC cells (Chen et al., 2015). In addition, in the upstream mechanism, POU3F3 (Zeng et al., 2020) overexpression activated the invasion and migration of cancer cells via miR-30d-5p, which was obviously 2021associated with the depth of tumor invasion and the degree of lymph node metastasis in esophageal carcinoma. Zhu et al., 2017; Zheng et al., 2021 illustrated a new lncRNA/Pseudogene-hsa–miR-30d-5p–GJA1 regulatory network associated with pancreatic cancer metastasis, and found that CTNNA1, CTNNB1 and CTNND1 were likely to participate in this network. In gallbladder cancer cells, LDHA activity inhibited cellular activities (He et al., 2018). After FC-11 treatment, LDHA activity was decreased and the invasion of tumor cells was also significantly reduced. MiR-30d-5p directly combined with the 3′-UTR site of LDHA, hence the imbalance of miR-30d-5p affected the movement and invasion of tumor cells. Yu and Liu, (2020) proposed that miR-30d-5p prevented the growth of ovarian granulose cells and facilitated apoptosis, while the overexpression of Smad2 reversed this effect.

### Cell Autophagy and Apoptosis

As supported by experiments, autophagy is closely related to cancer cell metastasis (Qin et al., 2015). Autophagy has been linked to ROS and mercaptan REDOX status (Desideri et al., 2012; Peiris-Pagès et al., 2015). In hepatocellular carcinoma cells, GLDC regulated the autophagy and invasion of cells via silting miR-30d-5p (Zhuang et al., 2019). Therefore, GLDC overexpression inhibited intrahepatic tumor metastasis. In cholangiocarcinoma, LDHA silencing promoted apoptosis through miR-30d-5p/LDHA axis (He et al., 2018). In addition to its prominent role in cancer, miR-30d-5p is involved in the autophagy process in brain injury. Jiang et al., 2018 found that exosomes loaded with miR-30d-5p reversed ischemia-induced autophagy-mediated brain damage via facilitating M2 microglia polarization, which is a feasible direction towards mitigating brain injury. Moreover, a recent study reported that various miRNAs, especially miR-30d-5p, were downregulated in brain hypoxia-ischemia (HI) (Wu et al., 2018). miR-30d-5p targeted the 3′-UTR site of Beclin1 mRNA and participated in autophagy in a newborn rat HI brain via regulating Beclin1. The C2dat2/miR-30d-5p/DDIT4/mTOR axis formed a new signaling pathway facilitating autophagy induced by cerebral ischemia reperfusion injury (Xu et al., 2021). Specifically, C2dat2 blocked the targeting function of miR-30d-5p on DDIT4, and promoted the upregulation of DDIT4 and Beclin-1 levels.

### Tumorigenesis

Tumorigenesis and tumor development may occur due to various reasons (Karki and Kanneganti, 2019), with the abnormal expression of oncogenes widely believed to be the principal cause (Tan et al., 2020; Jiang et al., 2021; Saito et al., 2021). Many studies reported that miRNAs are involved in tumor development under the action of oncogenes. Relevant studies revealed that PVT1 is an oncogene (Yu et al., 2018) that facilitates the evolvement of colon cancer though the miR-30d-5p/RUNX2 axis. The level of RUNX2, the downstream target of miR-30d-5p, was consistent with the expression trend of PVT1 in tumor tissues. *In vitro*, LOC440173 competitively sponged miR-30d-5p (Wang et al., 2020). Furthermore, HDAC9, a target gene of miR-30d-5p (Cheetham et al., 2013), was found to participate in genetic epigenetic modification through the deactivation of acetylated lysine on histones, and to play a carcinogenic role in ESCC (Figure 1). In addition, the occurrence of malignant conjunctival melanoma (Larsen, 2016) in the Danish population was epidemiologically associated with the upregulation of miR-30d-5p. In MDS samples, the expression of miR-30d-5p was significantly reduced, and the target was concentrated in the AML pathway (Ozdogan et al., 2017), indicating that miR-30d-5p is connected with tumor development, but the specific mechanism has not been clarified. LAMC3 might be regulated by 15 miRNAs, including miR-30d-5p, to affect the motility of cancer cells (He et al., 2019). What’s more, miR-30d-5p reduced the expression of ADAM12 promoted by LINC00284, which significantly inhibited the progression of thyroid malignant disease (Hu C. et al., 2021). In lung cancer, miR-30d-5p also had the function of reducing tumor occurrence and development, which depended on the ceRNA regulatory mechanism of circCD151/miR-30d-5p/GLI2 (Zhao et al., 2021). Taken together, researches into miR-30d-5p in tumorigenesis have progressed, while the specific mechanism needs further investigations.

### Effect of Drugs

In the cell experiments of non-small cell lung cancer, the application of cryptomatrone increased miR-30d-5p expression and limited the metastatic ability of lung cancer cells, suggesting that it might be a therapeutic direction to delay the progression of the disease, but more researches were still needed (Wang et al., 2019a). In addition to the above effects of miR-30d-5p, we summarized the related impacts of different drugs on cell activities involving miR-30d-5p and the participating pathways. Resveratrol (Res) is a polyphenol whose food sources include wine, berries and peanuts. This compound has many beneficial properties, such as anticancer and anti-aging effects (Galiniak et al., 2019). The protective role of Res was established in H9C2 cells through the miR-30d-5p/SIRT1/NF-JB axis in hypoxia-induced apoptosis (Han et al., 2020b). But this mechanism was just reported in cardiac protection, and it would be interesting to explore whether it would be present in malignancies.

## Conclusion

Many scholars have suggested that miRNA imbalance had a significant influence on the occurrence and progression of human cancers. This review summarized the key effects of miR-30d-5p in different types of tumors, such as lung cancer, colorectal cancer, prostate cancer, and esophageal cancer, emphasizing the vital roles of this potential biomarker of human cancers. MiR-30d-5p has been proven to be associated with clinical indications. When the expression of miR-30d-5p is detected to be significantly different from that of healthy tissue, it may indicate that miR-30d-5p is an significant factor in the diagnosis of tumorigenesis. Meanwhile, the up-regulation or down-regulation of miR-30d-5p during tumor development may also reflect the prognosis of cancer. Even though the relationship between miR-30d-5p and clinical indications is helpful to the prognosis of cancer, the specific mechanism of miR-30d-5p in tumors still deserves to be clarified. In particular, the relationship between miR-30d-5p in some cancers, such as ovarian cancer, uterine cancer and thyroid cancer, needs more researches to explore. In summary, miR-30d-5p is considered an important target for further studies. We highlight its potentially significant role in other cancers, as well as its ability to become a target and biomarker in the diagnosis and treatment of a variety of human cancers. To this end, research efforts targeting the miR-30d-5p-related mechanisms are expected.
